# Neuroprotective properties of curcumin in toxin-base animal models of Parkinson’s disease: a systematic experiment literatures review

**DOI:** 10.1186/s12906-017-1922-x

**Published:** 2017-08-17

**Authors:** Xin-Shi Wang, Zeng-Rui Zhang, Man-Man Zhang, Miao-Xuan Sun, Wen-Wen Wang, Cheng-Long Xie

**Affiliations:** 1 Department of Neurology, The First Affiliated Hospital of Wenzhou Medical University, Wenzhou, 325000 China; 2 The center of Traditional Chinese Medicine, The Second Affiliated Hospital and Yuying Childrens Hospital of Wenzhou Medical University, Wenzhou, 325027 China; 3 The center of rehabilitation, The First Affiliated Hospital of Wenzhou Medical University, Wenzhou, 325000 China

**Keywords:** Curcumin, Parkinson’s disease, Preclinical studies, Animal models, Systematic review

## Abstract

**Background:**

Curcumin (diferuloylmethane), a polyphenol extracted from the plant *Curcuma longa*, is widely used in Southeast Asia, China and India in food preparation and for medicinal purposes. Meanwhile, the neuroprotective actions of curcumin have been documented for experimental therapy in Parkinson’s disease (PD).

**Methods:**

In this study, we used a systematic review to comprehensively assess the efficacy of curcumin in experimental PD. Using electronic and manual search for the literatures, we identified studies describing the efficacy of curcumin in animal models of PD.

**Results:**

We identified 13 studies with a total of 298 animals describing the efficacy of curcumin in animal models of PD. The methodological quality of all preclinical trials is ranged from 2 to 5. The majority of the experiment studies demonstrated that curcumin was more significantly neuroprotection effective than control groups for treating PD. Among them, five studies indicated that curcumin had an anti-inflammatory effect in the PD animal models (*p* < 0.05). Meanwhile, four studies showed the antioxidant capability of curcumin, by which it protected substantia nigra neurons and improved striatal dopamine levels. Furthermore, two studies in this review displayed that curcumin treatment was also effective in reducing neuronal apoptosis and improving functional outcome in animal models of PD. Most of the preclinical studies demonstrated the positive findings while one study reported that curcumin had no beneficial effects against Mn-induced disruption of hippocampal metal and neurotransmitter homeostasis.

**Conclusions:**

The results demonstrated a marked efficacy of curcumin in experimental model of PD, suggesting curcumin probably a candidate neuroprotective drug for human PD patients.

## Background

Parkinson disease (PD), a chronic progressive neurodegenerative disorder predominantly affecting elderly people, is mainly characterized by resting tremor, rigidity, bradykinesia, postural instability and gait disorder [1]. It is supposed that about 6 to 10 million people worldwide have suffered from PD, involving all ethnicities and races [2]. The quantity of individuals affected by PD increases rapidly with age, affecting approximately 1% of the population over sixty years and 4% of those over eighty years [3]. As the mean age of the population rises, the prevalence of individuals worldwide is predicted to be doubled by 2030 [4]. At present, supplemental dopamine remains the primary therapy way that can adequately improve functional capacity and quality of life in PD [2]. Nevertheless, most patients develop related motor complications after five years of administration, including L-dopa-induced dyskinesia and motor fluctuations [5]. Surgical techniques like deep brain stimulation (DBS) can improve advanced symptoms and presentations and is regarded to exceed the best medical therapy. But, regrettably, less than 5% of the PD subjects may be qualified for such operations [5]. Consequently, there has been an emerging interest in the use of novel therapeutic strategies and natural antioxidants or plant molecules with neuroprotective characters are being developed for add-on therapy for PD patients.

Curcumin (diferuloylmethane) is widely used in Southeast Asia, India and China in food preparation or for medical purposes [6]. Moreover, it shows anti-oxidant, anti-inflammatory and anti-cancer features. It crosses the blood-brain barrier and is neuroprotective in central neurological disease [7]. Among them, the most pivotal biological function of curcumin related to neuroprotection is its anti-oxidant effect, which can guards substantia nigra (SN) neurons and increases striatal dopamine count and chelates Fe^2+^ in the 6-OHDA (6-hydroxydopamine) rat models of PD [8]. What is more, consequent to its anti-oxidant activity, curcumin also exerts mitochondrial protection in various PD models. Recently, several studies in different experimental models of PD have showed neuroprotective effect of curcumin. For instance, one study had recently demonstrated that chronic dietary consumption of turmeric offers neuroprotection in toxic mouse model of PD [9]. Wang et al. reported that curcumin administration restored mitochondrial membrane potential, increased Cu/Zn SOD (Superoxide Dismutase) and restored cell viability in 6-OHDA-lesioned MES (mouse embryonic stem) 23.5 cells [10]. Similarly, Rajeswari and colleagues had showed an increase in striatal dopamine and DOPAC (3,4-Dihydroxyphenylacetic acid) levels following curcumin injection in MPTP (1-methyl-4-phenyl-1, 2,3,6-tetrahydropyridine) injected mice [11]. To some extent, animal experiments may give insights into the mechanisms of PD, but a single study can not fully uncover all the details. Moreover, no clinical studies have tested the benefits of curcumin in PD patients. Hence, the exquisite benefits of curcumin in human PD are not well understand at present. The aim of this review was to systematicly describe the therapeutic potential of curcumin in animal models of PD and hope to provide more comprehensive assessment of the effects.

## Methods

We implemented this systematic review based on the modified Preferred Reporting Items for Systematic Reviews and Meta-Analyses (PRISMA) Statement.

### Search strategy

A carefully literatures search was performed to seek publications studying the effect of curcumin treatment on animal models of PD from Google scholar, PubMed, Chinese National Knowledge Infrastructure (CNKI), Wanfang and VIP information database. The time is from the inception of individual database up to February 2016 for all English or Chinese language publications. The following search strategy was used for each database.

1. Curcumin
2. Turmeric
3. Carcuma Longa
4. or/1–3
5. Parkinson’s disease
6. Parkinson disease
7. PD
8. or/5–7
9. 4 and 8

In this study, two experienced reviewers (WWW and ZRZ) selected the qualified studies independently by browsing the abstracts or full texts based on the eligibility criteria. Divergences were resolved by consensus with a third party (CLX).

### Inclusion and exclusion criteria

Inclusion criteria were pre-established as the following:

1. Controlled researches assessing in vivo administration of curcumin to animals with PD;
2. Laboratory animals of any species, age, gender, or strain to induced PD models were included; (3) Any kind of curcumin intervention that was compared with placebo control was included. Formulation, dosage, route of treatment, and curcumin therapy time were not limited;
3. Original data being independent from other studies and no outcome measure restrictions were imposed.

Pre-established exclusion criteria were including:

1. Case reports, editorials, abstracts, reviews, letters, end game or comments et al.;
2. Not testing the efficacy of curcumin on PD models;

### Data extraction and quality assessment

Two authors separately performed data collection, with disputes resolved by analysis and discussion. The detailed information from each study was listed as following: (1) Publication year, the first author name and experimental models; (2) Individual data were acquired from each animal, including number, weight, species, sex, anesthetic used, method used to induce PD models et al.; (3) Finally, route of administration, duration of treatment and dosage and outcome measures were also excerpt. We assessed the risk of bias of the included basic researches by applying a six-item modified scale according to our previous study [12].

## Results

### Results of the search

**Fig. 1:**
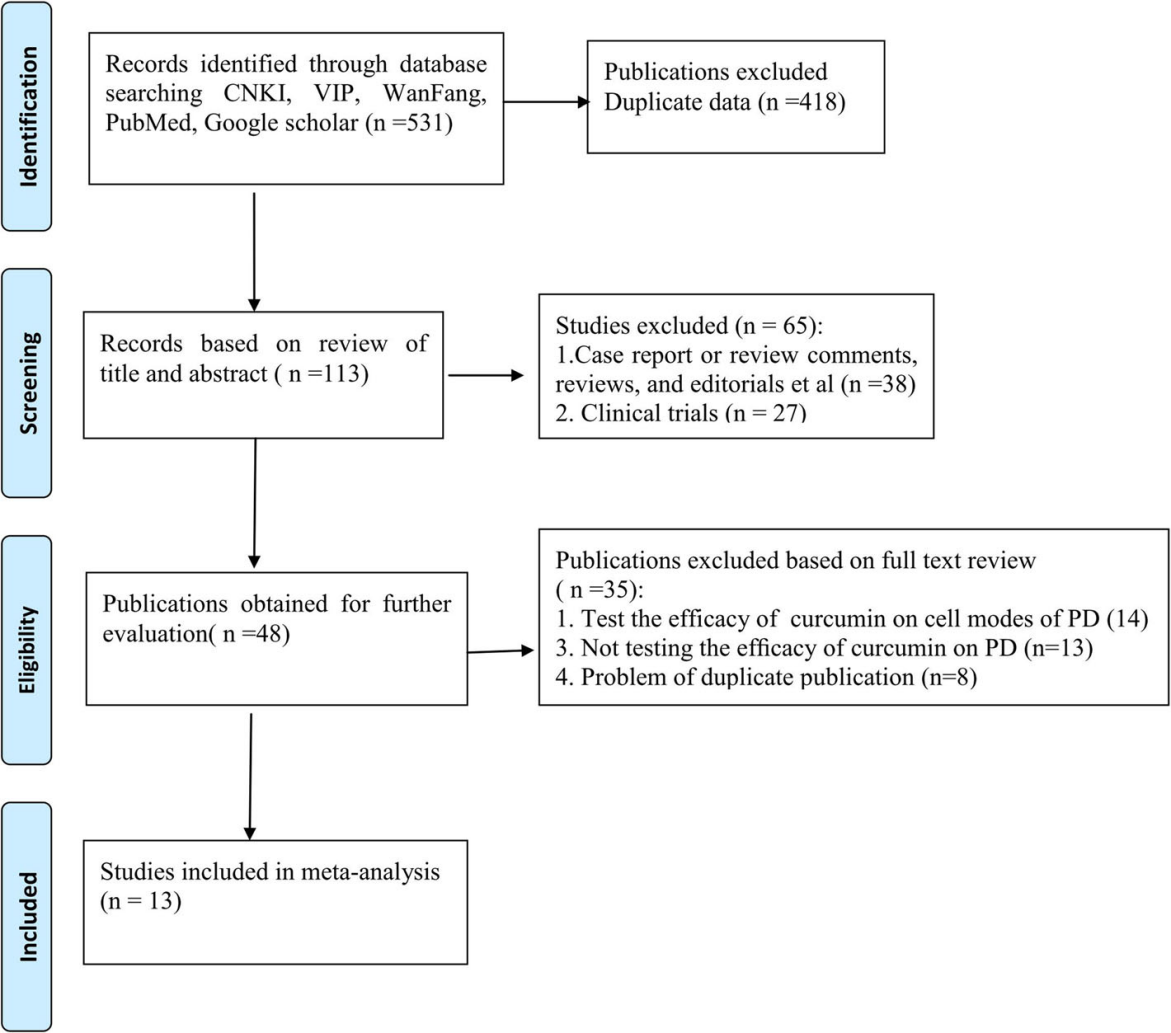
Algorithm of study selection and inclusion in the meta-analysis

Based on our searches of the electronic databases and after removing reproduction we identified a total of 113 references. After looking through the titles and abstracts, we eliminated 65 papers with at least one of following reasons: (1) Case report, comments, reviews, or editorials; (2) Human trials. And finally, after reading the whole text of the remaining 48 studies which reported the efficacy of curcumin in animal models of PD, we incorporated 13 articles and assessed these for eligibility [11, 13–24] (Fig. 1).

### Study characteristics

In this review, 13 studies involved 298 animals (Curcumin 173, control 125) from two species and five varieties: C57BL/6 mice (*n* = 6),Sprague-Dawley rats (*n* = 2), Swiss albino mice (*n* = 2), Wister rats (*n* = 2) and ICR (Imprinting Control Region) strain mice (*n* = 1). The studies varied in size, involving 12–40 animals. The rats and mice weighted 200–300 g and 20-55 g, respectively. Twelve studies utilized male animals and the remaining one used the female rats to perform the experiments. Five out of thirteen studies (38.4%) were 1-methyl-4-phenyl-1,2,3,6-tetrahydropyridine (MPTP) models, and other four studies (30.7%) utilized 6-OHDA lesioned models. The remaining four studies were using BSO (Buthionine sulfoximine) induced, homocysteine injected intracerebroventricularly, lipidosome induced and inhalation Mn induced PD models, respectively. Meanwhile, pentobarbital were used in 3 studies (23.1%), 10% Chloral hydrate in 2 study (15.4%), isofiuorane in 1 study (7.7), while no report of anesthetics in the remaining 7 studies (53.8%). Two studies performed a dose gradient of curcumin in the included studies, of which one study adopt 5, 50, 150 mg/kg dissolve in 1% DMSO (intraperitoneally, i.p.) and the other one utilized 500 and 1500 PPM (Parts per million). Unfortunately, most studies did not show the curcumin purity in the paper. Meanwhile, in this review, 7 studies showed curcumin to be dissolved in DMSO, 4 studies in saline, 1 study in cremophor, and the remaining 1 to be supplemented in the diet (food pellets). Moreover, the method of IHC (Immunohistochemistry) or IFC (Immunofluorescence) was used as the molecular biology techniques in 7 studies, HPLC (High-performance liquid chromatography) analysis in 5 studies, western blot in 5 studies, and RT-PCR (Reverse transcription-polymerase chain reaction) in 2 studies in this review. To our knowledge, IHC and HPLC were probably used to investigate the neuroprotective effects whereas techniques of Western blotting and RT-PCR were likely used to investigate the potential mechanisms involved in the neuroprotective effects. The basic characteristics of the 13 studies are shown in Table 1.

**Table 1:** Basic information of included studies

Study (years)	Species (Sex,n)	Model, anesthetic, weight	Interventions	Research methods
Virginia 2005	SD Rats (Male,6/6)	6-OHDA induced (12μg, s.i.), Isofiuorane,200 g	Curcumin (50 mg/kg dissolve in 10% of Cremophor, gavage) for 4 days prior to lesioning	1. IHC 2. HPLC analysis
Pan 2007	C57BL/6 mice (Male,30/10)	MPTP induced (30 mg/kg, i.p.), Pentobarbital, 20 g	Curcumin (5, 50, 150 mg/kg dissolve in 1% DMSO, i.p., respectively) for 4 days after surgery	1. IHC 2. WB analysis
Balusamy 2008	C57BL/6 mice (Male,6/6)	BSO induced (3 mmol/kg, i.p.), NR, 25 g	Curcumin (50 mg/kg dissolve in saline, i.p., bw per day, for 1 and 3 days)	1. WB analysis
Rajeswari 2008	Swiss albino mice (Male, 8/8)	MPTP induced (40 mg/kg, i.p.), NR, 25–30 g	Curcumin (80 mg/kg dissolve in DMSO, i.p.) for 7 days after surgery	1. HPLC analysis
Wang 2009	C57BL/6 mice (Male,10/10)	MPTP induced (60 mg/kg, i.p.), NR, 25–30 g	Curcumin (15 mg/kg dissolve in DMSO, i.p.) for 4 days prior to lesioning and after surgery	1. RT-PCR 2. HPLC analysis
Song 2010	C57BL/6 mice (Male,10/10)	MPTP induced (30 mg/kg, i.p.), NR, 23–25 g	Curcumin (50 mg/kg dissolve in DMSO, i.p.) for 5 days after surgery	1. Behavioral tests and WB 2. IHC
Peng 2010	SD Rats (Male,20/20)	6-OHDA induced (16μg, s.i.), Pentobarbital, 200–250 g	Curcumin (100 mg/kg dissolve in DMSO, gavage) for 4 weeks after surgery	1. Behavioral tests 2. HPLC analysis
Zahra 2012	Wister rats (Male,8/8)	Homocysteine induced (2 mol/l, i.c.v.), NR, 250–300 g	Curcumin (50 mg/kg dissolve in saline, i.p.) for 10 days beginning 5 days prior to Hcy injection	1. Behavioral tests 2. IHC and Tunel staining
Du 2012	Wister rats (Female,12/12)	6-OHDA induced (20μg, s.i.), Chloral hydrate, 200–220 g	Curcumin pretreatment (200 mg/kg dissolve in saline) twice a day for 24 days in total	1. HPLC analysis 2. IHC
Yu 2012	C57BL/6 mice (Male,10/10)	MPTP induced (30 mg/kg, i.p.), NR, 25 g	Curcumin (50 mg/kg dissolve in saline, i.p.) for 5 days after surgery	1. Behavioral tests and WB 2. IHC and iron staining
Guo 2012	C57BL/6 mice (Male,30/10)	Lipidosome induced (1 μg/ml, s.i.), Chloral hydrate, 18-22 g	Curcumin (10, 20, 40 mg/kg dissolve in 1% DMSO, respectively, i.p.) for 4 days after surgery	1. Behavioral tests and WB 2. Elisa, RT-PCR, IFC 3. Luciferase assay
Tripanichkui 2013	ICR strain mice (Male,7/7)	6-OHDA induced (33μg, s.i.), Pentobarbital, NR	Curcumin (200 mg/kg dissolve in DMSO, i.p.) for 7 days after surgery	1. IHC 2. Quantification Kits
Ariana 2014	Swiss albino mice (Male, 16/8)	Inhalation Mn induced, NR, 45–55 g	Curcumin (500 or 1500 PPM) was supplemented in the diet for 14 weeks	1. Behavioral tests 2. Neurochemical Analysis

*MPTP* 1-methyl-4-phenyl-1, 2,3,6-tetrahydropyridine; *SD Rats* Sprague-Dawley rats; *BSO* Buthionine sulfoximine; *6*-*OHDA* 6-hydroxy dopamine; *NR* no report; *s*.*i*. Striatum injection; *i*.*p*. intraperitoneally; *i*.*c*.*v*. intracerebroventricularly; *DMSO* Dimethyl sulfoxide; *PBS* Phosphate-buffered saline; *IHC* Immunohistochemistry; *HPLC* High-performance liquid chromatography; *WB analysis* western blot; *RT*-*PCR* Reverse transcription-polymerase chain reaction; *IFC* immunofluorescence; *Elisa* the enzyme-linked immunosorbent assay

### Risk of bias

The risk of bias of included studies were ranged from 2 to 5 out of a total 6 points. Two studies got 2 points (15.4%); six studies got 3 points (46.1%); four studies got 4 points (30.7%); one study got 5 points (7.8%). The risk of bias of the 13 studies are shown in Table 2.

**Table 2:** Risk of bias of included studies

Study	Virginia 2005	Pan 2007	Balusamy 2008	Rajeswar 2008	Wang 2009	Song 2010	Peng 2010	Zahra 2012	Du 2012	Yu 2012	Guo 2012	Tripanichkui 2013	Ariana 2014
A	√	√	√	√	√	√	√	√	√	√	√	√	√
B	√				√			√	√	√	√	√	√
C	√												
D													
E	√	√	√	√	√	√	√	√	√	√	√	√	√
T	√		√	√		√		√	√			√	√
Total	5	2	3	3	3	3	2	4	4	3	3	4	4

A: peer reviewed publication; B:random allocation to group; C: blinded assessment of outcome; D: a sample size calculation; E: compliance with animal welfare regulations; F: a statement of a potential conflict of interest

### Neuroprotective mechanisms of curcumin

**Fig. 2:**
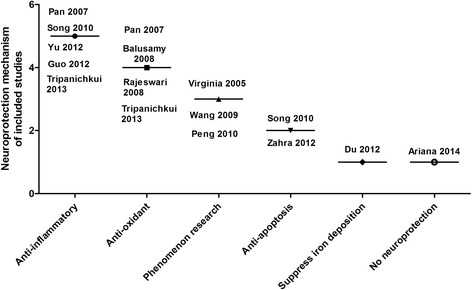
Neuroprotection mechanisms for curcumin in treating Parkinson’s disease

Table 3 showed the main outcome measures and results of included studies. TH-positive (Tyrosine hydroxylase) cells or TH mRNA level in substantia Nigra was tested in 7 studies; striatal concentration of DA (Dopamine) and its metabolites DOPAC and HVA (Homovanillic acid) were inspected in 6 studies. Meanwhile, four studies investigated GFAP (Glial fibrillary acidic protein) expression in the striatum as an indicator of astrocyte activation; two studies tested TNF-a (Tumor necrosis factor) level and iron-deposition, respectively. Moreover, one study showed the levels of GSH (Glutathione), ROS (Reactive oxygen species), MAO-B (Monoamine Oxidase-B) activity, NF-kβ, IL-1b (Interleukin), SOD1 and metal Mn, separately. Fig. 2 showed the neuroprotective mechanism of curcumin (Fig. 2).

**Table 3:** Main outcome measures of included studies

Study (years)	Outcome measures	Neuroprotection mechanism
Virginia 2005	1. TH-positive cells in the SN were increased by curcumin. 2. Striatal concentration of DA and its metabolites DOPAC and HVA	Phenomenon research.
Pan 2007	1. TH-positive cells and protein level in the SNpc and striatum. 2. GFAP-positive cells and iNOS level in the SNpc	Anti-oxidant and anti-inflammatory.
Balusamy 2008	1. Curcumin against GSH depletion-mediated oxidative stress, significantly restored total brain GSH levels in BSO mice. 2. Accumulation of ROS was prevented only by pretreatment with curcumin.	Anti-oxidant capabilities.
Rajeswari 2008	1. Curcumin reversed the reduction in striatal DA and DOPAC levels; 2. MAO-B activity was reduced by curcumin treatment	Anti-oxidant capabilities.
Wang 2009	1. Curcumin reversed the reduction in SNpc TH and DAT mRNA levels; 2. DA and DOPAC levels were restored by curcumin	Phenomenon research.
Song 2010	1. Curcumin showed a significant increase in locomotion frequencies; 2. Curcumin increased the TH, DAT level and inhibits astrocyte activation in terms of GFAP. 3. Inhibitory effects of curcumin on JNK, c-Jun, and caspase-3.	Anti-inflammatory and anti-apoptosis.
Peng 2010	1. Curcumin could ameliorate rotational behaviour; 2. DA level was restored by curcumin administration.	Phenomenon research.
Zahra 2012	1. Curcumin prevented the decrease of locomotor activity. 2. The number of Nissl neurons on the left side of substantia nigra was significantly higher in curcumin group. 3. Effect of curcumin on Bax/Bcl-2.	Anti-apoptosis.
Du 2012	1. Curcumin partly restored the levels of DA, DOPAC and HVA. 2. TH-positive neurons were restored by curcumin pretreatment. 3. Marked decrease of iron-positive cells was found in the curcumin pretreatment group.	Suppress the iron-induced degeneration.
Yu 2012	1. Curcumin ameliorated open-field test; 2. TH, DAT levels were restored by curcumin and inhibited GFAP and TNF-a.	Anti-inflammatory.
Guo 2012	1. Motor coordination of rota-rod test and hanging test were improved in the curcumin treatment group. 2. Curcumin suppressed nuclear translocation and NF-Kβ activity. 3 TNF-a and IL-1b were restored by curcumin.	Anti-inflammatory
Tripanichkui 2013	1. Curcumin attenuated loss of TH fibers, diminished activation of GFAP and microgliosis, sustained SOD1 level.	Anti-inflammatory and anti-oxidant
Ariana 2014	1.Curcumin produced similar deleterious effects in the beam-walking test and single-pellet test. 2. Curcumin showed no beneficial effects against Mn-induced disruption of hippocampal metal and neurotransmitter homeostasis (DA or serotonin).	No neuroprotection

*TH* Tyrosine hydroxylase; *SN* Substantia Nigra; *DA* dopamine; *DOPAC* 3,4-Dihydroxyphenylacetic acid; *HVA* Homovanillic acid; *iNOS* Inducible nitric oxide synthase; *GFAP* Glial fibrillary acidic protein; *GSH* Glutathione; *ROS* Reactive oxygen species; *MAO*-*B* Monoamine oxidase-B; *DAT* dopamine transport; *JNK* c-Jun N-terminal kinase; *SOD* Superoxide Dismutase; *TNF*-*α* Tumor necrosis factor; *IL*-*1β* Interleukin-1β

### Curcumin exhibites anti-inflammatory property

Five studies indicated that curcumin had anti-inflammatory effect on the PD animal models. Pan et al. [14], Yu et al. [21] and Tripanichkui et al. [23] reported that curcumin could diminished the GFAP level compared with control group (*p* = 0.002). Yu S et al. [17] demonstrated that MPTP -treated mice exhibited 2.5 times more activated astrocytes than the control mice (*p* < 0.01). MPTP plus curcumin treatment obviously relieved the MPTP-induced increase in the amount of activated astrocytes (*p* < 0.01). Moreover, Guo et al. [22] displayed that curcumin suppressed nuclear translocation and NF-kβ activity, as well as reduced the levels of TNF-a and IL-1b (*P* < 0.05).

*Curcumin is used as an antioxidant*

Four studies showed the anti-oxidant function of curcumin which protected SN neurons and improved striatal dopamine levels. Pan et al. [14] reported iNOS level in the SNpc was obvious reduced by curcumin treatment compared with MPTP group (*p* = 0.005). Jagatha B et al. [15] demonstrated the neuroprotective fumnction of curcumin against GSH depletion-mediated oxidative stress in the cerebra. Intraperitoneal injection of curcumin resulted in a 2-fold increase in total brain GSH levels. Meanwhile, accumulation of ROS was avoided by pretreatment with curcumin. Rajeswari et al. displayed a significant decrease in MAO-B activity in the striatal region by curcumin treatment. Guo et al. [22] demonstrated that 6-OHDA significantly decreased SOD1 expression in the ipsilateral striatum (*p* < 0.05), which was impeded by curcumin (*p* < 0.05).

*Curcumin treatment is effective in decreasing neuronal apoptosis and suppressing iron-deposition*

In this review, two studies showed that curcumin administration was effective in decreasing neuronal

apoptosis as well as improving functional outcome in animal models of PD. Immunoblotting results of Yu S et al. [17] showed that MPTP induced an apparently increase in phosphorylated forms of JNK. Interestingly, curcumin administration (50 mg/kg for 7 days) overtly inhibited MPTP-induced JNK phosphorylation. Meanwhile, Zahra et al. [19] demonstrated Bax/Bcl2 ratio significantly increased in the control group, which was apparently reduced by curcumin administration (*p* < 0.001). Du et al. [20] reported that the quantity of iron-positive cells raised in the lesioned side compared with control. However, a striking decrease of iron-positive cells was discovered in the curcumin pre-treatment group, compared to the 6-OHDA-lesioned group. Interestingly, only one study [24] showed that curcumin had no helpful effects against Mn-induced disruption of hippocampal metal and neurotransmitter homeostasis. Future studies are needed to confirm these results and investigate if other mechanisms are involved in Mn-induced experimental model of Parkinsonism.

## Discussion

Thirteen preclinical trials with a total of 298 animals were included in the analysis. Our results indicated that, comparing with the placebo group, curcumin could improve the neurobehavioral function and restore the levels of TH, DA, DOPAC et al., suggesting that curcumin offers neuroprotection in animal models of PD probably via antioxidant capabilities, anti-inflammatory and anti-apoptosis. To our knowledge, there is no doubt that curcumin is helpful in the treatment of animal models of PD, but we are not aware of whether animal researches reliably inform human studies. Further evidence is necessary in this area by assessing curcumin in clinical trials. However, we should also note that the poor bioavalibility of curcumin will limit its clinical application.

### Neuroprotective mechanisms of curcumin in treating PD

#### Curcumin exhibites anti-inflammatory property

**Fig. 3:**
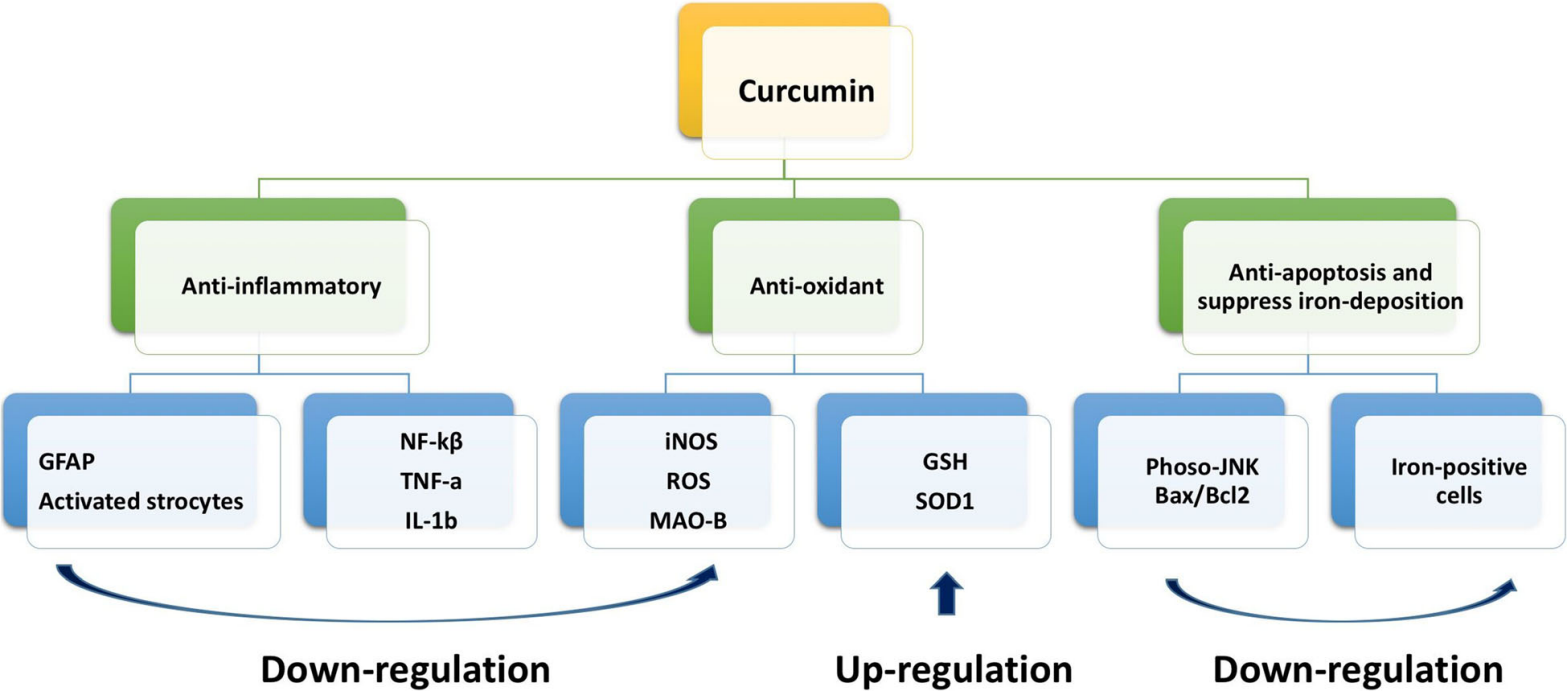
Schematic representation the neuroprotective mechanisms of curcumin in treating PD. Curcumin was shown to improve neurological function and exhibited anti-oxidant, anti-inflammatory and anti-apoptosis properties, as a neuroprotective agent used in PD

As shown in Fig. 3 that neuroprotection mechanisms for curcumin in treating PD are various (Fig. 3). Pre-treatment or post-treatment of curcumin in 6-OHDA-lesioned rats resulted in reduced DA neuron loss compared with placebo group and attenuated the loss of DOPAC and HVA acid in the striata [13]. Exactly, the neuroprotective function of curcumin against PD is related to its anti-oxidant capability. MES cells treated with curcumin remarkably augmented the expression of Cu-Zn superoxide dismutase and decreased intracellular ROS accumulation [10]. Furthermore, accumulation of oxidative DNA damage has been uncovered in PD and transition metal ions such as Cu and Fe powerfully inhibit the DNA repair enzymes [25]. However, curcumin can changeover such inhibition of DNA repair enzymes both in neuroblastoma cells and in vitro [26]. Meanwhile, Rajeswari et al. showed that DA depletion and elevated monoamine oxidase-B activity, as a function of MPTP-induced toxicity, was alleviated with curcumin. Obviously, the most important biological function of curcumin pertinent to neuroprotection is its antioxidant function.

#### Curcumin is used as an antioxidant

Treatment of curcumin in PD animal models evidently attenuated the loss of DA neurons in SN. The potential effects of curcumin on NO generations and associated signaling pathways in LPS-induced cell PD models had been preliminary investigated [27]. Additionaly, curcumin could weaken the generation or viability of prostaglandins, glutamate, and pro-inflammatory cytokines in rat’s hypothalamus and reduce the levels of GFAP, a marker of astrocytic proliferation [28]. What is more, curcumin restored mitochondrial membrane potential and modulated NF-Kβ nuclear translocation by inhibition of IL-6 and TNF-a [29]. Meanwhile, curcumin treatment abrogated dopamine-induced striatal neuron cell death by prevention of c-Jun phosphorylation and caspase 3 activation [17].

#### Curcumin treatment is effective in reducing neuronal apoptosis

Jaisin et al. depicted that the level of p53 was down-regulated in SH-SY5Y cell lines using a 6-OHDA-lesioned model by curcumin administration. The protection gained by curcumin treatment against 6-OHDA toxicity was because of the suppression of p53 phosphorylation and the restoration of balance between anti- and pro-apoptotic proteins of Bcl-2 family [30]. One study have explicitly demonstrated that the molecular mechanism involved in the neuroprotection of curcumin against MPP + −mediated apoptosis in PC12 cells is via Bcl-2 signal pathway and decreased levels of pro-apoptotic proteins, Bax and Bad [31]. The survival rate of cells being treated with 0.25 mmol/L curcumin was 72% compared with 45% in MPTP alone. What is more, Yu and colleagues showed that curcumin suppressed JNK-mediated dopamine neuron apoptosis induced by MPP+ exposure in both SH-SY5Y cell models and C57BL/6 mice [17]. Previous findings suggested that in addition to anti-oxidant and anti-inflammatory features, the function of curcumin in PD also is related to complex regulatory of cell-mediated apoptosis such as JNK pathway.

#### Assessment of the risk of bias

To our knowledge, there is no established valid and reliable tool to evaluate the risk of bias in animal studies, so the risk of bias assessments of each systematic review is inconsistent. To help overcome barriers in the switching of preclinical trials to clinical studies, the original Stroke Therapy Academic Industry Roundtable publication gave a recommendation for the preclinical development of acute ischemic stroke (AIS) [32]. Although recognized to be rational, they have not been closely followed or rigorously validated. Therefore, updated new and appropriate preclinical recommendations were urgent [33]. Though it was a recommendation for basic researches of supposed acute stroke therapies, the modified six-items from the suggestions could be regarded as a criterion for the risk of bias of animal studies for other models [34]. The new and amended preclinical suggestions may provide a basis for further consideration and careful discussions [33]. Overall, we believe that the standard suggestions were helpful in improving many features of preclinical testing. Meanwhile, one need realized that fulfilling them does not guarantee success in clinical development. Nonetheless, rigorous and thorough preclinical design could provide reassurance that there is potentially a greater chance for success in clinical trials.

#### Implication for further studies

Animal researches are an essential early step toward evaluating and developing an intervention for clinical trials in humans [35]. Systematic reviews have been supposed to be vital for translating the findings from preclinical researches to human studies. We believe that similar technique can be used to increase our comprehending of sources of bias in animal experiments as used in clinical trial, which will result in improvements in study quality [36]. To our knowledge, curcumin is a well-known drug for the treatment of PD researches in vitro. Although the present evidence is inadequate to support efficacy of curcumin in clinic, it is a promising candidate for furture PD patients. Nevertheless, sub-therapeutic levels resulted from low bioavailability of orally curcumin continues to be the main hindrance of curcumin administration and poses great challenges to date. Meanwhile, various studies have demonstrated that curcumin is insoluble in aqueous solution, extremely unstable in alkaline condition, and very easily degraded and metabolized by human body. In this paper, 4 studies used saline to dissolve is unsuitable. Therefore, future researches with animal need to select suitable solvents. Several studies reported that the conversion of water-insoluble curcumin into nano-sized particles or being dissolved in DMSO could greatly improve curcumin’s solubility in vivo. In addition, other plant-derived polyphenols are also increasingly receiving attention as dietary supplements for the homeostatic management of central nervous system disorders. Similarly, the poor bioavailability of some polyphenols (such as silybin, green tea or proanthocyanidin) likely contributes to poor clinical trial. Based on this situation, in the future, more studies should focus on investigating new ways to improve bioavailability of curcumin and other polyphenols.

#### Interpretation of the results

Curcumin is a natural product with multiple biological function and plentiful potential therapeutic applications in neurological diseases [37]. In addition, more efforts are needed to make out how and why curcumin can have these pharmacological effects, taking into consideration its low bioavailability. By means of analyzing the similarities between the biological function of curcumin and its degradation products against central nervous disorders, it appears that the degradation products may contribute to the pharmacological effects of curcumin [38]. Meanwhile, recently, one research suggested that the degradation products should play a pivotal role in the diverse biological activities of curcumin [39]. This new finding not only offer novel insights into the complex pharmacology of curcumin due to its poor bioavailability, but also provide new approach for developing therapeutic applications of this natural product. Whatever, low systemic bioavailability of curcumin due to its low aqueous solubility and poor stability is a main disadvantage, which has severely limited its clinical application. To overcome this obstacle, experts have recently tried strategies to improve its bioavailability through various delivery systems including nanoparticles, liposomes, micelles, etc. [40]. Improved bioavailability, by various delivery systems or other means, is expected to bring improved bioefficacy in clinical application.

## Conclusions

In this systematic review, curcumin, as a neuroprotective agent used in PD, exhibited anti-oxidant, anti-inflammatory and anti-apoptosis properties, and was shown to improve neurological function. Several studies in different experimental models of PD strongly support the clinical trial application of curcumin in PD patients. However, we still need more well-designed RCTs (Randomized controlled clinical trials) to ensure the efficacy of curcumin in PD patients.

## Abbreviations

6-OHDA: 6-hydroxydopamine
BSO: buthionine sulfoximine
DA: dopamine
DMSO: dimethylsulfoxide
DOPAC: 3,4-Dihydroxyphenylacetic acid
GFAP: glial fibrillary acidic protein
GSH: Glutathione
HPLC: high-performance liquid chromatography
HVA: homovanillic acid
ICR: imprinting Control Region
IFC: immunofluorescence
IHC: immunohistochemistry
IL-1b: interleukin
MAO-B: monoamine oxidase-B
MES: mouse embryonic stem
MPTP: 1-methyl-4-phenyl-1,2,3,6-tetrahydropyridine
PD: Parkinson’s disease
ROS: reactive oxygen species
RT-PCR: reverse transcription-polymerase chain reaction
SN: substantia nigra
SOD: superoxide dismutase
TH: tyrosine hydroxylase
TNF-a: tumor necrosis factor
